# The relationship between frontal alpha asymmetry and behavioral and brain activity indices of reactive inhibitory control

**DOI:** 10.1152/jn.00046.2024

**Published:** 2024-06-12

**Authors:** Atakan M. Akil, Renáta Cserjési, Tamás Nagy, Zsolt Demetrovics, Dezső Németh, H. N. Alexander Logemann

**Affiliations:** ^1^Doctoral School of Psychology, ELTE Eötvös Loránd University, Budapest, Hungary; ^2^Institute of Psychology, ELTE Eötvös Loránd University, Budapest, Hungary; ^3^Institute of Psychology, Faculty of Humanities and Social Sciences, University of Pécs, Pécs, Hungary; ^4^Centre of Excellence in Responsible Gaming, University of Gibraltar, Gibraltar, Gibraltar; ^5^College of Education, Psychology and Social Work, Flinders University, Adelaide, South Australia, Australia; ^6^INSERM, Université Claude Bernard Lyon 1, CNRS, Centre de Recherche en Neurosciences de Lyon CRNL U1028 UMR5292, Bron, France; ^7^NAP Research Group, Institute of Psychology, Eötvös Loránd University & Institute of Cognitive Neuroscience and Psychology, Research Centre for Natural Sciences, Budapest, Hungary; ^8^Department of Education and Psychology, Faculty of Social Sciences, University of Atlántico Medio, Las Palmas de Gran Canaria, Spain; ^9^Department of Clinical, Neuro and Developmental Psychology, Vrije Universiteit Amsterdam, Amsterdam, Netherlands

**Keywords:** event-related potentials, frontal alpha asymmetry, reactive inhibitory control, stop signal task, transcranial direct current stimulation

## Abstract

Reactive inhibitory control plays an important role in phenotype of different diseases/different phases of a disease. One candidate electrophysiological marker of inhibitory control is frontal alpha asymmetry (FAA). FAA reflects the relative difference in contralateral frontal brain activity. However, the relationship between FAA and potential behavioral/brain activity indices of reactive inhibitory control is not yet clear. We assessed the relationship between resting-state FAA and indicators of reactive inhibitory control. Additionally, we investigated the effect of modulation of FAA via transcranial direct current stimulation (tDCS). We implemented a randomized sham-controlled design with 65 healthy humans (M_age_ = 23.93, SD_age_ = 6.08; 46 female). Before and after 2-mA anodal tDCS of the right frontal site (with the cathode at the contralateral site) for 20 min, we collected EEG data and reactive inhibitory performance in neutral and food-reward conditions, using the stop signal task (SST). There was no support for the effect of tDCS on FAA or any indices of reactive inhibitory control. Our correlation analysis revealed an association between inhibitory brain activity in the food-reward condition and (pre-tDCS) asymmetry. Higher right relative to left frontal brain activity was correlated with reduced early-onset inhibitory activity and, in contrast, linked with higher late-onset inhibitory control in the food-reward condition. Similarly, event-related potential analyses showed reduced early-onset and enhanced late-onset inhibitory brain activity over time, particularly in the food-reward condition. These results suggest that there can be a dissociation regarding the lateralization of frontal brain activity and early- and late-onset inhibitory brain activity.

**NEW & NOTEWORTHY** This research reveals dissociation between baseline frontal alpha asymmetry and the timing of reactive inhibitory brain activities in food-reward contexts. Whereas inhibitory control performance decreases over time in a stop signal task, electrophysiological indices show reduced early- and heightened late-onset inhibitory brain activity, especially in the reward condition. Additionally, greater right frontal activity correlates with reduced early-onset and increased late-onset inhibitory brain activity.

## Graphical abstract

**Figure:**
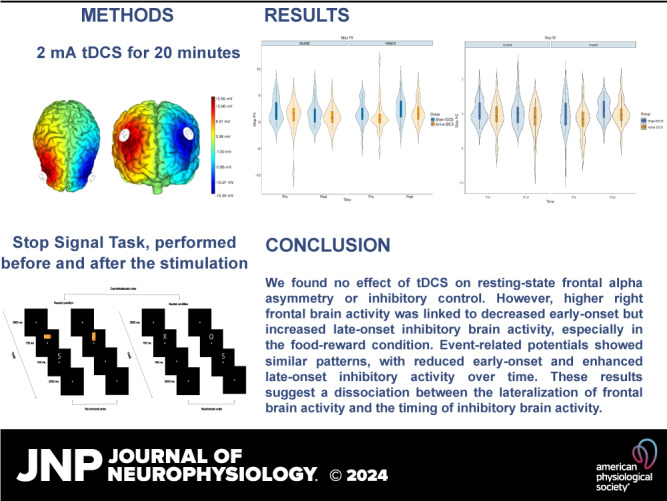

## INTRODUCTION

Reactive inhibitory control, the ability of individuals to withhold a prepotent response subsequent to a stop signal, contributes to adaptive functioning (1, 2), and its failure has been associated with conditions such as obesity (3), obsessive-compulsive disorder (4, 5), and Parkinson’s disease (6). Previous studies have suggested that a dominance of relative right frontal cortical activity over the left is presumably linked to the inhibitory system, whereas left dominance is associated with the approach system (7–9). In particular, frontal alpha asymmetry (FAA), which is an electrophysiological (EEG) measure of the difference in alpha power (8–13 Hz) between the left and right frontal activity, may serve as a useful marker of inhibitory control. One of the candidate generators (at least in part) of approach and avoidance systems is the right dorsolateral prefrontal cortex (DLPFC) activity asymmetry (9–12), which may conversely be associated with upregulation of avoidance and downregulation of approach motivations in the inhibitory system. However, the exact relationship between FAA and specific indicators of reactive inhibitory control triggered as a function of intrinsic reward (e.g., food contexts) is not yet clear. The main aim of the present study was to address this open question.

Individual differences in reactive inhibitory control are commonly assessed with the stop signal task (SST) (13, 14). In this task, participants are presented with “go” stimuli (in this case palatable food pictures, the items that motivate individuals to approach them) at the center of the screen, requiring a button-press response. Occasionally, these go stimuli are followed by a “stop” stimulus (a specific symbol), signaling participants to withhold their response. The stop-signal reaction time (SSRT), a widely accepted measure of reactive inhibitory control, is derived from this task. Previous studies utilized a combination of the SST and event-related potential (ERP) analyses to explore brain mechanisms underlying inhibitory control (15–17). ERPs reflect the synchronous activation of large groups of neurons time-locked to an event (18). The Stop N2, occurring at ∼200-ms latency, exhibits significantly more negative amplitudes during successful stop trials than during unsuccessful ones (13). Furthermore, some research suggested that the neurobiological correlate of the Stop N2 is the right inferior frontal gyrus (13). The Stop P3 is influenced by stopping success, showing larger amplitudes for successful inhibitions compared to failed ones (13). Indeed, the Stop P3 has been thought to represent inhibitory control (19) and to originate from the superior frontal gyrus (20). Despite these presumed associations, reactive inhibitory control’s behavioral and EEG indices have not been explored in relation to FAA.

We employed transcranial direct current stimulation (tDCS), which is a noninvasive neuromodulation technique that delivers electrical currents to the brain through electrodes placed on the scalp to modulate specific brain activities (21). Promisingly, various studies have provided some support indicating that tDCS over DLPFC (specifically, anode over right DLPFC) reduces cravings, desire to binge eat, and overall food intake in both clinical and nonclinical samples such as in individuals with eating disorders (22–24), even though cathodal stimulation effects outside the right prefrontal cortex are still not understood (25). Furthermore, Kekic et al. (23) indicated that tDCS effects may last hours after administration, even after a single tDCS session. However, despite some evidence regarding the effects of tDCS over DLPFC on reactive inhibitory control, to the best of our knowledge it is still unknown whether these effects can be attributable to changes in resting-state FAA.

Based on the literature, we employed a food condition in addition to a neutral condition, which would make the effect of tDCS more sizable. Another reason for this was that related research found that there is a distinction between learned reward (e.g., money) and intrinsic reward (e.g., palatable foods that contain fat and sugar), which is associated with challenged inhibitory control (26). We hypothesized that a reduction of FAA scores, induced by 2-mA active tDCS (anode over right DLPFC/cathode over left DLPFC) for 20 min, would be associated with increased reactive inhibitory control within the food context as opposed to the neutral context. This increase of reactive inhibitory control was expected to be manifested as a decreased SSRT in the SST, along with greater amplitudes in the Stop N2 and Stop P3.

## MATERIALS AND METHODS

### Participants

Our pilot study (*n* = 10, with 9 right-handed participants) regarding the main effect of tDCS on FAA helped in determining the sample size of the main study. We used G*Power (27, 28) with a defined power of 80%, alpha at 0.05, and estimated FAA test-retest correlation of 0.6. The expected smallest effect [*f* > 0.237; partial η^2^ ($\eta_{p}^{2}$) > 0.053] was estimated to be detectable with the a priori intended sample size of 30 for the active intervention group. Sixty-five healthy humans (46 females, 57 right-handed), ranging in age from 18 to 58 yr (M_age_ = 23.93 yr; SD_age_ = 6.08), were ultimately recruited via social media and university courses for the study after several participants terminated the experiment for a variety of reasons, such as boredom and fatigue. To participate in the study, individuals needed to meet certain eligibility criteria. Specifically, they had to be at least 18 yr of age. Additionally, participants were screened for exclusion criteria, which included the presence of psychological or psychiatric disorders, frequent headaches or migraines, metallic implants, epilepsy, significant head trauma in the past, recent head trauma, pacemaker usage, chronic skin conditions, and current drug use. They also avoided smoking and drinking coffee at least 2 h before the experiment. Previous research found that handedness does not affect reactive inhibitory control (29). Therefore, we did not consider handedness as an exclusion criterion. PsyToolkit was used for self-report assessments (30, 31). Before any procedures took place, all participants provided their written informed consent. The research was conducted following the ethical guidelines outlined in the Declaration of Helsinki and its later amendments. The study was approved by the local research ethics committee (protocol number: 2020/403). Each participant was given a voucher or course credit for their participation.

### Stop Signal Task

**Figure 1.**
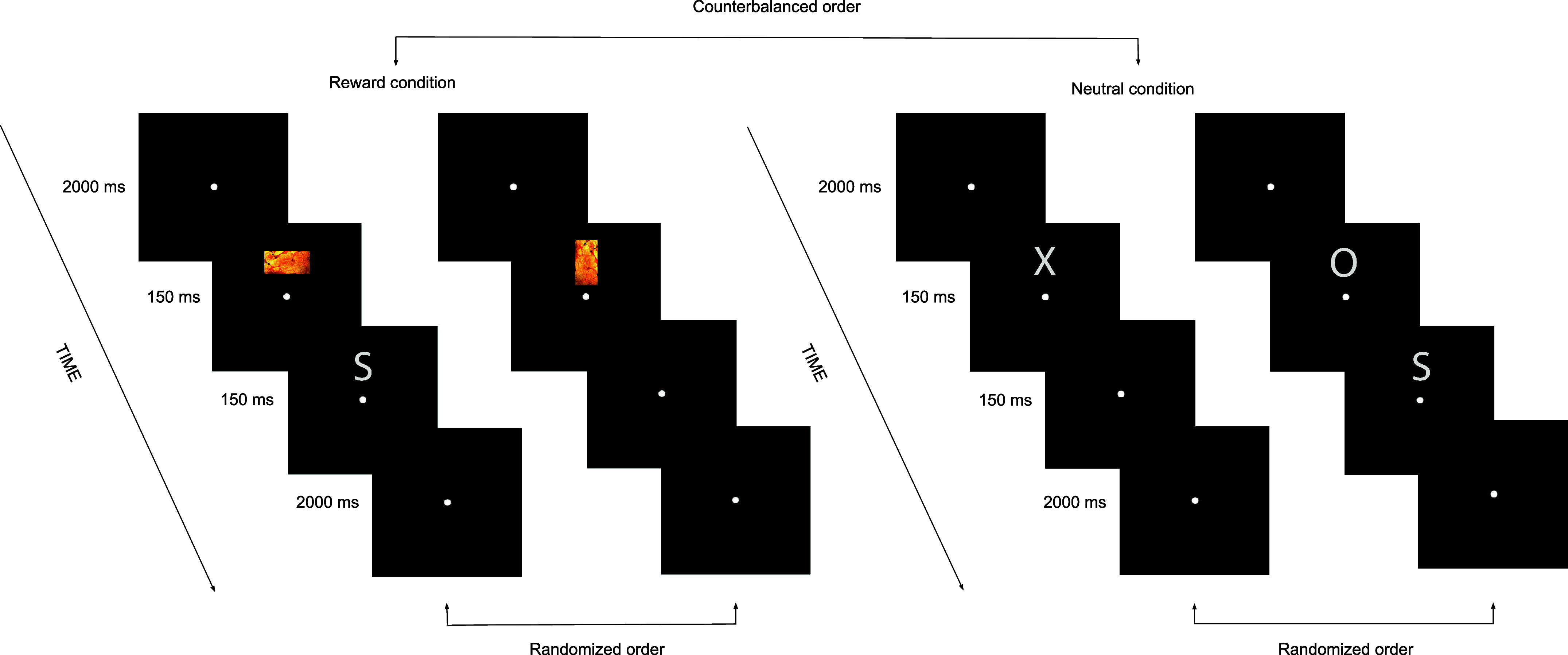
Based on the counterbalanced order, participants started the stop signal task (SST) under either the neutral or food-reward condition. Per condition, a practice block with stop trials was used for estimating optimal go-stop delay to start with in the subsequent first experimental block. The overall experimental design involved 1,536 go trials and 512 stop trials. The initial 2 trials (on *left*) represent the food-reward condition, where the target dimension (horizontal/vertical) and the palatable food pictures (such as chips, chocolate, cookies, nuts, etc.) were randomly presented for 150 ms. The final 2 trials (on *right*) represent the neutral condition, where the letters “X” and “O” were randomly presented for 150 ms. These trials were sometimes followed by the letter “S” indicating stop trials, where participants should withdraw their initiated responses to nonstop stimuli displayed beforehand.

The SST was developed with OpenSesame (32). Figure 1 illustrates the task. It was adapted from the original version (13) and included a food-reward condition based on previous studies (26, 33). Per condition, a practice block with stop trials was used for estimating optimal go-stop delay to start with in the subsequent first experimental block. A total of four experimental blocks were implemented per condition, with each block containing 96 go trials and 32 stop trials, constituting 25% of the total trials; therefore, each block contained 128 trials in total. We conducted 512 trials per condition. Each phase of the study (i.e., either premodulation or postmodulation) consisted of 1,024 in total, including both neutral and food-reward conditions. Consequently, the overall experimental design consisted of 2,048 trials, including two phases and 16 blocks (8 for each phase) but excluding practice blocks. Participants were seated ∼65 cm from the screen. At the beginning of the task, participants were initially asked to read written instructions, and verbal clarification was provided upon request. First, a fixation dot was displayed in the middle of the screen to engage attention and eye fixation for 2,000 ms. Slightly above the dot, in the food-reward condition palatable food pictures, i.e., cookies, chips, chocolate, and nuts [115 px width (2.9°) × 200 px height (5.1°)], which required a key response, were presented randomly in horizontal or vertical orientation for 150 ms. Therefore, the food-reward condition had two dimensions (horizontal and vertical). On the other hand, in the neutral condition instead of the palatable food pictures, a letter “X” or “O” [200 px width (5.1°) × 200 px height (5.1°)] was presented for 150 ms, which also required a key response depending on the letter presented. Either a left button press or a right button press with the left and right index fingers based on a stimulus was used for responses. In stop trials, go stimuli (either palatable food pictures in horizontal or vertical position or letter X or O depending on the condition) were infrequently followed by the letter “S” [200 px width (5.1°) × 200 px height (5.1°)] for 150 ms representing “stop,” and participants were supposed to withhold their response in that case. The stop signal delay was fixed at 350 ms, but when participants could not withhold their response, the timing was arranged after each stop trial via a staircase algorithm; the go-stop delay was decreased 50 ms. In case of a successful inhibition, the go-stop delay increased 50 ms. The tracking algorithm yields an ∼50% inhibit rate, increasing the reliability of the estimation of the SSRT (34). In total, a session of the task took ∼45 min. The main outcome of the SST was the SSRT. Following Verbruggen et al. (34), omissions were replaced with maximum reaction times (RTs) (1,500 ms).

### EEG Acquisition

Scalp voltages were captured with a 21-channel cap Ag/AgCl electrode set according to the 10-20 system. The brand was Mind Media NeXus-32 (35). The vertical electrooculography (VEOG) was recorded above and below the left eye, and the horizontal EOG (HEOG) was recorded bipolarly from the outer canthi of both eyes. Sampling rate was set at 512 Hz. EEG was continually recorded except when tDCS was applied.

### Frontal Alpha Asymmetry

FAA scores were derived from EEG data recorded during two separate 5-min resting-state sessions, one with eyes open (EO) and another with eyes closed (EC), both before and after the intervention. Collecting EEG resting-state recordings under these two circumstances facilitates a clearer grasp of the effects of sensory input, internal cognitive functions, and the inherent activity of the brain. This approach contributes to a thorough comprehension of the functional organization of the brain in various states (36). With BrainVision Analyzer 2 (www.brainproducts.com), FAA was calculated based on previous reports (37). First, a high-pass filter of 0.5 Hz, a low-pass filter of 40 Hz, and a 50-Hz notch filter were applied. Subsequently, the first and last 10 s of the EEG data was excluded because of artifacts. The data were then segmented into 2-s epochs. Based on VEOG and HEOG electrodes, the epochs for the EO condition were adjusted for ocular artifacts by independent component analysis (ICA). Epochs that still contained artifacts, determined by a 75-µV maximum amplitude +/− relative to the baseline criterion, were excluded. The power spectral density (PSD) was determined by fast Fourier transform (FFT) with a 10% Hanning window for the remaining epochs after whole segment baseline correction. Next, the epochs were averaged, and the mean activity in the alpha frequency band (8–13 Hz) was calculated. Values for the relevant electrodes were exported. With SPSS 22 (38), alpha power was corrected for skew via natural log transform (37). Finally, frontal asymmetry was calculated by subtracting the log-transformed alpha at lateral left electrode sites from right electrode sites (i.e., F4 − F3 and F8 − F7).

### Event-Related Potentials

BrainVision Analyzer 2 (www.brainproducts.com) was used for preprocessing. First, signals were referenced to linked mastoids. Subsequently, following previous studies [Logemann et al. (39, 40)], EEG data were filtered (offline) with a high cutoff of 30 Hz, a low cutoff of 0.5 Hz, and notch filter of 50 Hz. Data were segmented into epochs ranging from −100 ms to 2,600 ms. Ocular corrections were conducted by independent component analysis. After that, epochs were segmented and they were baseline corrected with the baseline set at −100 to 0 ms. We conducted a go-signal locked segmentation, then baseline correction followed by artifact rejection (using minimal/maximal allowed amplitude −75 µV/75 µV and marking 200 ms before and after events as bad). We employed a stop-signal locked segmentation and baseline correction. Next, we computed separate averages for segments corresponding to failed stops and successful stops. The inhibitory ERPs were computed by subtracting the average stop-signal locked activity for failed stops from successful ones. Following a thorough examination of the grand average waveforms, we have identified specific latency intervals for further analysis. Previous studies have shown that the N2 modulation by stopping success is most pronounced at right frontal sites (FC4/FC2) and the P3 modulation at frontocentral sites (FCz/Cz) [e.g., Schmajuk et al. (13), Logemann et al. (16)]. For the Stop N2 component, we extracted data from the time window of 166–286 ms at F4, a nearby site, since our EEG setup did not include FC4/FC2 electrode sites. For the Stop P3 component, data from the 211–271 ms time window at Cz were used for export.

### Transcranial Direct Current Stimulation

**Figure 2.**
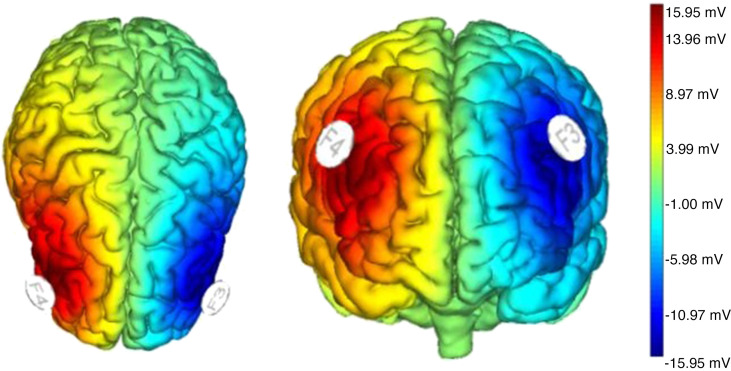
This illustration displays the positioning of the transcranial direct current stimulation electrodes and the protocol applied. To administer transcranial direct current stimulation, a steady current of 2 mA was passed through 2 circular sponge electrodes (25 cm^2^ each), positioned on the scalp over locations F4 (anode) and F3 (cathode) with the use of a saline solution. It was applied for 20 min. The maximum strength of the electric field reached 15.95 µV at the anodal electrode.

The aim of brain modulation was to modulate frontal alpha asymmetry. A pair of circular sponges (25 cm^2^) soaked in saline solution were used to deliver direct electrical current with STARSTIM-8 (Neuroelectrics; www.neuroelectrics.com). According to the 10-20 system, the anode was positioned on the right DLPFC (F4) and the cathode on the left DLPFC (F3). Unless it was a sham condition, a steady current of 2 mA was applied for 20 min [Kelley et al. (9)] (Fig. 2). The safety of this parameter has been shown in healthy subjects (41). In the sham condition, a brief current was also applied to make it comparable sensation-wise to the active condition. Upon completion of the experiment, participants were asked to identify the condition they had experienced. There was no statistically significant association between the actual group assignment and the correctness of participants’ guesses: χ^2^ = 0.08; df = 1; *P* = 0.772.

### Procedure

**Figure 3.**
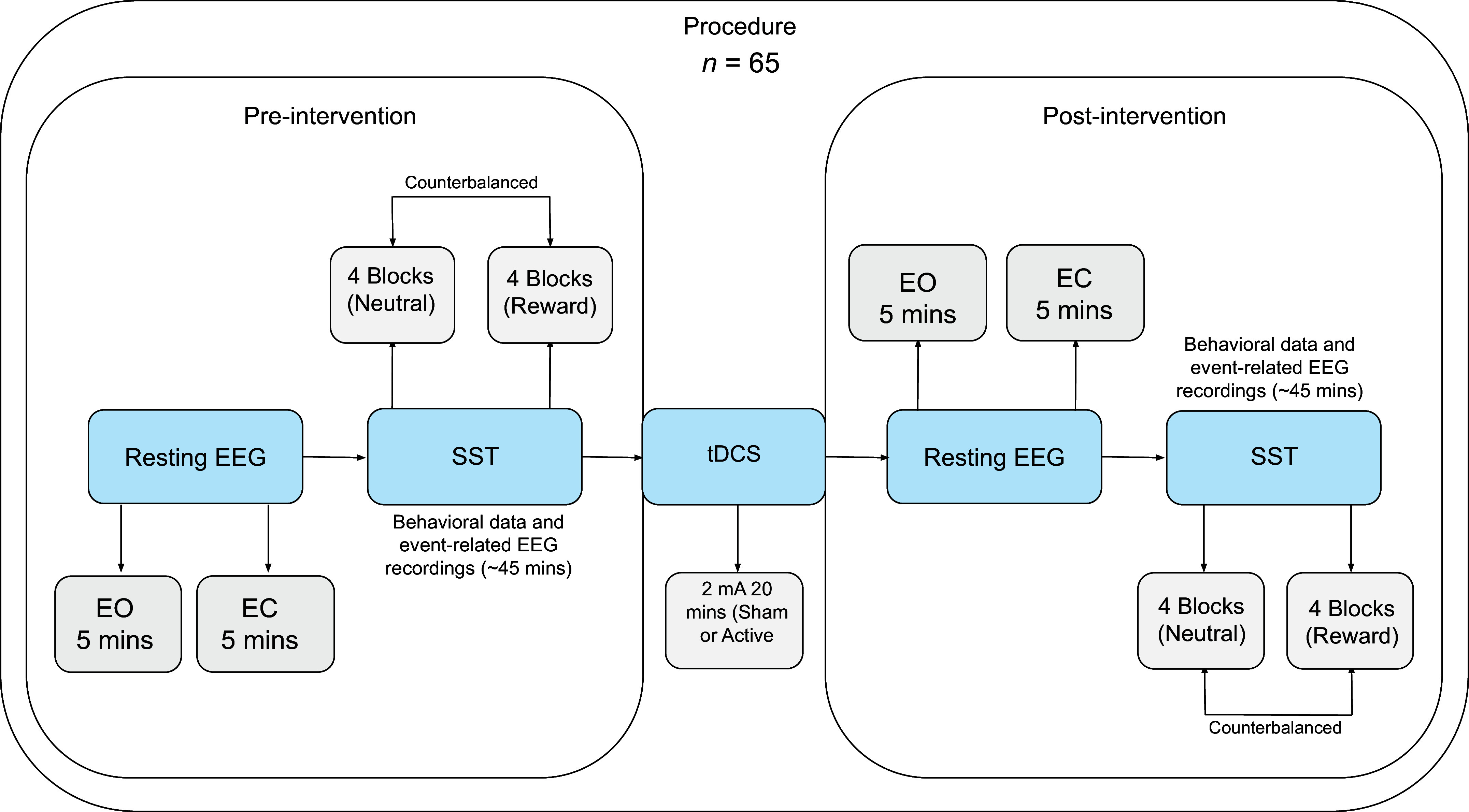
The experiment begins by gathering preintervention resting-state EEG data to measure frontal alpha asymmetry (FAA), which was conducted in 2 sessions: 1 with a 5-min period of eyes open (EO) and another with 5 min of eyes closed (EC) conditions. Subsequently, participants performed the stop signal task. After this, either sham or 2-mA active transcranial direct current stimulation (tDCS) was administered. In the sham condition, a brief current was also applied to make it comparable sensation-wise to the active condition. More details on the intervention can be found in Fig. 2. After the stimulation, the same procedure was repeated for the postmodulation assessment, including the resting-state EEG and the stop signal task (SST).

The study was preregistered on Open Science Framework (https://osf.io/6k8up/?view_only=0cfdcc8e92f441e0905a456611a78762). A randomized, triple-blind, sham-controlled design was used in this research, with within-subject (time: pre/postassessment, condition: neutral/food-reward) and between-subject (group: active/sham tDCS) factors (see Fig. 3).

Before and after the neural modulation session, the SST was completed. Participants were seated in a comfortable chair in a dimly lit testing room for the placement of EEG electrodes on the scalp sites after reading the information letter, verifying the inclusion and exclusion criteria, and completing the informed consent form when they arrived at our laboratory. Subsequently, resting-state EEG data were collected, recording for 10 min in total with two blocks of 5-min sessions (EO/EC). They finished the questionnaires after recording of the resting-state EEG. They then completed the first phase of the SST before the tDCS. EEG was recorded throughout the resting states and computer tasks but not during the intervention as the cap was changed. After that, individuals were assigned to either a 2-mA active or a sham tDCS for 20 min. After the intervention, participants immediately started the second phase of the experiment. During the postmodulation evaluation, the same steps, resting-state EEG and the SST, were repeated. The stimulation and pre/postassessments were conducted on the same day and lasted ∼5 h.

### Statistical Analyses

The analyses were conducted with SPSS 22 (38) and R (42). In addition to employing frequentist statistics, we also conducted a series of Bayesian analyses using JASP (43). Upon completing the calculations of the main variables, we excluded participants with missing values and outliers beyond 3 standard deviations (SDs) from the mean. Each model has a different number of participants excluded, and they are indicated in the tables. A mixed ANOVA with a 2 × 2 design was utilized to investigate hypotheses regarding FAA, whereas a 2 × 2 × 2 design was used for analyzing the behavioral and brain activity indices of reactive inhibitory control. We also conducted a correlation analysis to examine the relationship between baseline (pre-tDCS) FAA and behavioral and brain activity indices of reactive inhibitory control. Although previous research found that handedness does not affect reactive inhibitory control (29), as part of the exploratory analyses we excluded left-handed participants and examined the relationships. The results are provided in the Supplemental Materials. The electrophysiological variables investigated included FAA (EO/EC), the Stop N2, the Stop P3, in addition to the behavioral indices of inhibitory control, SSRTs. For all frequentist statistical analyses the significance level was set at 0.05, and for the null results a Bayesian approach was used with Bayes factor 01 (BF_01_), which is in favor of null hypotheses (H_0_) over alternative hypotheses (H_1_). More specifically, BF_01_ values ranging from 1 to 3 are indicative of anecdotal evidence, whereas values falling between 3 and 10 suggest substantial evidence in favor of the null hypothesis (H_0_). BF_01_ values exceeding 10 provide strong evidence for H_0_. Conversely, values ranging from 1 to 1/3 suggest anecdotal evidence against H_0_, whereas values between 1/3 and 1/10 indicate substantial evidence against H_0_. Values below 1/10 provide strong evidence against H_0_. Values around 1 do not support either hypothesis (44, 45). The results of Bayesian and exploratory analyses can be found in the Supplemental Materials.

## RESULTS

### Results regarding the Transcranial Direct Current Stimulation and Frontal Alpha Asymmetry

To examine the association between tDCS and resting-state FAA, a mixed ANOVA with time (pre/postintervention) as the within-subjects factor and group (active/sham tDCS) as the between-subject factor was conducted. We further performed a correlation analysis between baseline (pre-tDCS) FAA and behavioral and neural indices of reactive inhibitory control. The ANOVA results revealed no significant interaction between the time and group factors concerning FAA. Table 1 shows the details. These results were also supported by Bayesian statistics. We found substantial evidence in favor of the null hypothesis (BF_01_ > 3). Our exploratory analysis, limited to right-handed participants, similarly failed to yield any novel insights. For further information, see Supplemental Table S5.

**Table 1.** Results of FAA models

Variables	df	*F*	*P*	$\eta_{p}^{2}$	BF_01_
FAA F4 − F3 (EO) (*n* = 59)					
Group	1	3.64	0.058	0.030	1.7
Time	1	0.39	0.530	0.003	5.53
Group × time	1	0.28	0.594	0.002	8.85
Error	114				
FAA F4 − F3 (EC) (*n* = 59)					
Group	1	1.22	0.271	0.010	4.08
Time	1	1.27	0.261	0.010	3.64
Group × time	1	0.49	0.484	0.004	11.61
Error	114				
FAA F8 − F7 (EO) (*n* = 62)					
Group	1	<0.01	0.951	<0.001	6.30
Time	1	<0.01	0.933	<0.001	7.31
Group × time	1	0.29	0.588	0.002	27.49
Error	120				
FAA F8 − F7 (EC) (*n* = 59)					
Group	1	0.03	0.849	<0.001	5.57
Time	1	3.58	0.060	0.030	0.98
Group × time	1	0.08	0.769	<0.001	8.25
Error	114				

Participants with missing values and outliers (based on 3 SDs from the mean) were excluded from the analyses. BF_01_, Bayes factor 01; EC, eyes closed; EO, eyes open; FAA, frontal alpha asymmetry.

In the food-reward condition, we observed a significant negative correlation between baseline FAA F4 − F3 (EO) and two key brain activity indices of inhibitory control, namely the Stop N2 (*r* = −0.48, *P* < 0.001) and the Stop P3 (*r* = −0.34, *P* = 0.010). For further information regarding the correlation analysis, refer to Supplemental Table S6.

### Results regarding the Transcranial Direct Current Stimulation and Behavioral Indices of Inhibitory Control

**Figure 4.**
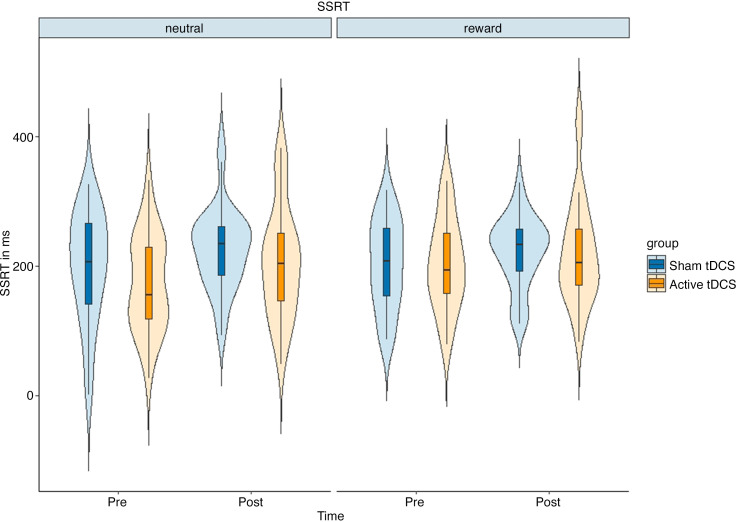
This figure displays the average stop-signal reaction times (SSRTs), considering the factors of time (pre/postintervention), condition (neutral/food-reward), and group [sham/active transcranial direct current stimulation (tDCS)]. The error bars indicate SEs. It illustrates that inhibitory control decreased from preassessment to postassessment, regardless of condition and group factors. It is important to note that longer SSRTs represent decreased inhibitory control.

To assess the relationship between tDCS and inhibitory control, we conducted a mixed ANOVA using time (pre/postintervention) and condition (neutral/food-reward) as within-subjects factors and group (active/sham tDCS) as the between-subject factor on SSRT scores. The results are presented in Fig. 4, and descriptive and inferential statistics are shown in Table 2 and Table 3, respectively. We found no significant effect of tDCS on the SSRT. However, there was a significant main effect of time on the SSRT, and it was supported by Bayesian statistics as well [*F*(1,220) = 6.33, *P* = 0.012, $\eta_{p}^{2}$ = 0.027, BF_01_ < 0.1]. These findings indicate that reactive inhibitory control decreases as time progresses, irrespective of the condition or group. As part of our exploratory analysis, left-handed participants were excluded. However, the new model did not reveal any new insights. Further details can be found in Supplemental Table S7.

**Table 2.** Descriptive statistics regarding the stop signal task performance

Variables (*n* = 57)	Min	Max	Mean	SD
*Preintervention*				
Neutral				
SSRT, ms	1	331.4	185.6	80.4
Go trials RT	339.7	926	647.2	125.6
Stop trials RT	237.7	442.9	307.7	42
Inhibition rate, %	10.9	57	46.3	8.1
Omission rate, %	0	44	8.7	9.6
Food-reward				
SSRT, ms	78.8	330.0	201.6	68.6
Go trials RT	377.7	973	666.8	132.1
Stop trials RT	441.9	436.6	308.7	
Inhibition rate, %	25.7	51.5	46.6	5.8
Omission rate, %	0	34.1	6.9	7.3
*Postintervention*				
Neutral				
SSRT, ms	48.3	389.2	215.8	75.9
Go trials RT	299.6	957.7	612	162.4
Stop trials RT	206.2	418.5	298.4	55.6
Inhibition rate, %	15.6	53.1	45.1	8.6
Omission rate, %	0	32.2	9	8
Food-reward				
SSRT, ms	82.6	430.9	220.6	68.5
Go trials RT	317.6	1015	616.6	155.9
Stop trials RT	224.5	473.6	301.2	57.3
Inhibition rate, %	13.2	54.6	45.6	7.8
Omission rate, %	0.2	36.7	9	8.4

The participant exclusion criteria included missing values, outliers, negative values, and inhibition rates under 10% for the calculation of stop-signal reaction times (SSRTs). RT, reaction time.

**Table 3.** Results of the stop signal task models

Variable (*n* = 57)	df	*F*	*P*	$\eta_{p}^{2}$	BF_01_
SSRT					
Time	1	6.33	0.012*	0.027	0.01
Condition	1	1.13	0.287	0.005	3.24
Group	1	1.49	0.222	0.006	4.28
Time × condition	1	0.33	0.565	0.001	3.94
Time × group	1	<0.01	0.956	<0.001	6.66
Condition × group	1	1.28	0.257	0.005	4.17
Time × condition × group	1	0.03	0.862	<0.001	5,591
Error	220				

Participants with missing values, outliers, negative values, and inhibition rates under 10% were excluded from the analysis. SSRT, stop-signal reaction time. *Statistically significant. BF_01_, Bayes factor 01.

### Results regarding the Transcranial Direct Current Stimulation and Brain Activity Indices of Inhibitory Control

**Figure 5.**
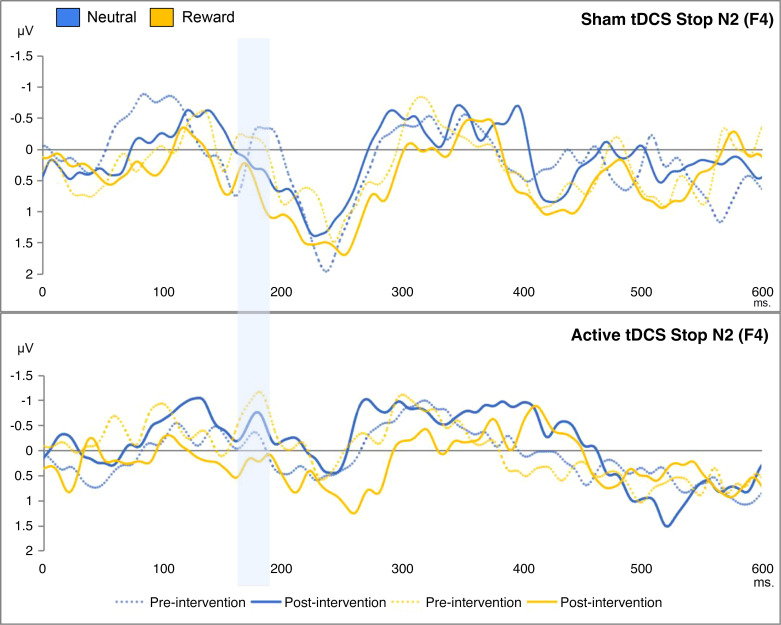
This figure shows the stop-signal locked event-related potentials during the stop signal task: effects of time, condition, and group on the Stop N2 (166–286 ms), based on successful inhibitions minus failed ones. The *x*-axes represent the time in milliseconds; the *y*-axes represent the Stop N2 scores in microvolts. The blue bar highlights the Stop N2 peaks. tDCS, transcranial direct current stimulation.

**Figure 6.**
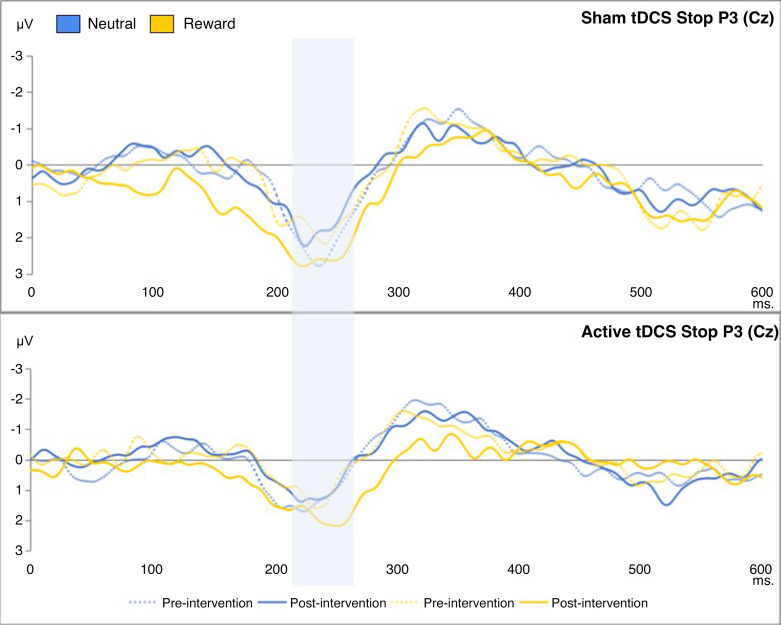
This figure shows the stop-signal locked event-related potentials during the stop signal task: the effects of time, condition, and group on the Stop P3 (211–271 ms), based on successful inhibitions minus failed ones. The *x*-axes represent the time in milliseconds; the *y*-axes represent the Stop P3 scores in microvolts. The blue bar highlights the Stop P3 peaks. tDCS, transcranial direct current stimulation.

**Figure 7.**
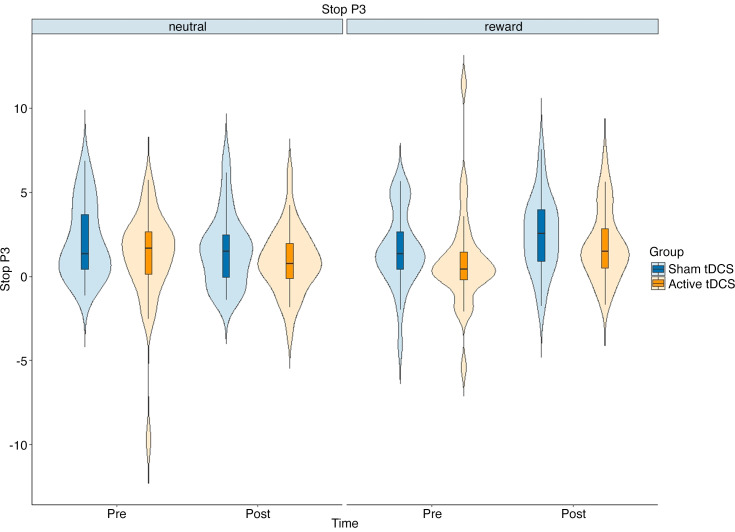
The figure shows the exact effect of time and condition interaction on the Stop P3. The *x*-axes represent the time factor; the *y*-axes represent the Stop P3 in microvolts. tDCS, transcranial direct current stimulation.

To evaluate the impact of the tDCS intervention on the brain activity measures of inhibitory control, we performed a mixed ANOVA. This analysis included time (pre/postintervention) and condition (neutral/food-reward) as within-subjects factors, and group (sham/active tDCS) was the between-subject factor. The focus was on the N2 and P3 amplitudes. The interaction effect of time, condition, and group on the indices was found to be insignificant. On the other hand, the previously reported Stop P3 and Stop N2 in the context of the SST were also evidenced in the current similar SST. They are displayed in Fig. 5 and Fig. 6, respectively. There was a statistically significant effect of the interaction between time and condition on the Stop P3 [*F*(1,228) = 7.18, *P* = 0.010, $\eta_{p}^{2}$ = 0.112, BF_01_ = 2.96], as illustrated in Fig. 7. In other instances, Bayesian factors supported H_0_ (BF_01_ > 3). More detailed results can be found in Table 4. As an exploratory analysis, we excluded the left-handed participants. Similarly, we found a significant effect of time and condition interaction on the Stop N2 [*F*(1,204) = 6.71, *P* = 0.010, $\eta_{p}^{2}$ = 0.031] (Supplemental Table S8). Supplemental Figure S8 indicated that, over time, there was an decrease in the early-onset inhibitory brain activity, particularly in the reward condition.

**Table 4.** Results of the event-related potential models

Variables	df	*F*	*P*	$\eta_{p}^{2}$	BF_01_
Stop N2 at 166–188 ms (F4) (*n* = 59)					
Time	1	1.25	0.264	0.005	8.80
Condition	1	0.29	0.591	0.001	14.62
Group	1	1.91	0.168	0.008	7.79
Time × condition	1	1.54	0.216	0.006	29.02
Time × group	1	0.20	0.649	<0.001	37.04
Condition × group	1	0.15	0.698	0.001	50.48
Time × condition × group	1	0.36	0.545	0.001	1008.24
Error	228				
Stop P3 at 211–271 ms (Cz) (*n* = 59)					
Time	1	0.80	0.329	0.003	6.05
Condition	1	0.97	0.323	0.004	5.56
Group	1	5.87	0.016*	0.025	3
Time × condition	1	4.21	0.041*	0.018	2.96
Time × group	1	0.10	0.750	<0.001	15.28
Condition × group	1	0.03	0.850	<0.001	15.50
Time × condition × group	1	0.04	0.825	<0.001	104.54
Error	228				

Participants with missing values, erroneous values, and outliers (based on 3 SDs from the mean) were excluded from the analyses. *Statistically significant. BF_01_, Bayes factor 01.

## DISCUSSION

Our study aimed to explore the association between FAA and behavioral and brain activity indices of inhibitory control. Contrary to our initial hypothesis, we did not observe any significant effect between tDCS and FAA, and there were also no noticeable impacts of tDCS on the behavioral and brain activity indicators of inhibitory control. A noteworthy trend emerged where SSRTs showed a decline across all conditions and groups over time, accompanied by a concurrent decrease in early-onset inhibitory brain activity and an increase in late-onset inhibitory brain activity in the intrinsic reward (food) condition. In our correlation analysis on the baseline (preintervention) frontal alpha asymmetry, greater right frontal brain activity compared to the left (indicating lower frontal alpha asymmetry) was found to be associated with reduced early-onset inhibition (as evidenced by Stop N2), yet it was associated with heightened late-onset inhibitory activity (as indicated by Stop P3).

Previous research has suggested a potential influence of tDCS on food-specific inhibitory control (23). However, these measures could be considered indirect because of the absence of comprehensive research into the direct relationship between frontal asymmetry and inhibitory control. One candidate measure for frontal asymmetry is the FAA. Nevertheless, we did not find any evidence for a relationship between FAA, the target of our tDCS application, and inhibitory control. Similarly, there was also no effect of tDCS and measures of reactive inhibitory control. The potential impact of tDCS on cognition and behavior is notably variable (46), challenging the notion of a polarity-specific influence. Although tDCS is theoretically expected to increase excitability under the anode and decrease it under the cathode, the actual cognitive and behavioral effects are far more complicated. Interestingly, some studies have even reported facilitatory effects associated with stimulation under the cathode, possibly attributed to noise reduction in specific networks, leading to improved performance (46). Alternatively, cathodal tDCS might inhibit a particular function as well, leading to enhanced performance in specific tasks, like faster reaction times (47). Numerous factors can contribute to these contradictory outcomes. For example, the effects of prefrontal tDCS heavily depend on the state of the targeted neural network (48). In the online paradigm, tDCS influences networks already engaged in the task, whereas in the offline paradigm, it modifies neural activity beyond the stimulation period. Understanding these state-dependent effects is crucial for cognitive and behavioral studies, as factors like fatigue, task knowledge, and network connectivity can significantly influence the baseline neural state. It should be noted that the exact electrode placement may also affect results. Specifically, results from a recent meta-analysis suggest that the effect on inhibitory control performance measures obtained in the SST and Go/No-go task may vary as a function of electrode placement, with more consistent results when the active electrode is placed over the right inferior frontal gyrus (49). Specifically, tDCS targeting the right inferior frontal gyrus demonstrated a medium effect size, whereas a stimulation site over the DLPFC region showed an overall null effect. Variation in results was attributed to the positioning of the return electrode, with extracephalic placement differing from various positions across the head. Factors related to electrode properties may have also played a role in shaping the outcomes. The size, shape, and conductivities of the electrodes, as well as the use of gels and saline solutions, may have influenced the distribution and intensity of the electric field (50). However, further studies are needed to further assess the potential moderating role of electrode placement and features.

Another plausible explanation could be attributed to the sample characteristics. Each participant’s unique brain structure, including factors like skull thickness and sulcal depth, could have resulted in divergent responses to tDCS (51–53). Research on binge eating (22–24) suggests that tDCS primarily affects inhibitory control in samples with considerable room for improvement. This hypothesis proposes that individuals with relatively weaker inhibitory control at baseline may experience more pronounced enhancements following tDCS intervention. Conversely, in healthy individuals the influence of tDCS may not predominantly target FAA and inhibitory activity but rather attentional control (54). Furthermore, frontal tDCS is known to produce more variable electric fields compared to other types of tDCS (55), adding further complexity to the neural adjustment processes. Our findings underscore the importance of considering individual differences and optimizing stimulation protocols in future research.

Our correlation analysis revealed a significant connection between inhibitory brain activity during food-reward conditions and baseline frontal brain asymmetry, as indexed by resting-state FAA before the neurostimulation. Precisely, greater right-sided frontal brain activity compared to the left side was linked to reduced initial inhibitory activity (Stop N2), likely emanating from the inferior frontal gyrus (13). However, it was also associated with heightened subsequent inhibitory control (Stop P3), which is thought to originate from the superior frontal gyrus (20). To the best of our knowledge, this is the first study showing the dissociation between baseline frontal EEG alpha asymmetry and the timing differentials of early- and late-onset reactive inhibitory brain activities within intrinsic reward contexts. However, some other research found simultaneous increase in N2 and P3 (56).

The interaction between different regions is crucial for complex processes. The observed correlation might suggest a dynamic relationship between the inferior and superior frontal gyri in managing inhibitory control. Higher activity in one region may trigger or facilitate inhibitory control processes in another region. Furthermore, the higher right frontal brain activity might signify a compensatory mechanism. When early-onset inhibitory activity originating from the inferior frontal gyrus is compromised, the brain might engage the superior frontal gyrus to enhance inhibitory control at a later stage. Alternatively, the nature and demands of the task being performed could influence how inhibitory control is exerted. Based on this, the brain’s inhibitory processes might operate differently at different stages of a task such as at early-onset inhibitory activity, associated with the inferior frontal gyrus, and late-onset inhibitory control involving the superior frontal gyrus to achieve optimal inhibitory control. It is important to note that the interpretation provided is speculative and would need to be validated through empirical research and neuroimaging studies.

Based on the SST results, a notable reduction in inhibitory control performance (i.e., increased SSRT) was observed as time progressed, which aligns with expectations due to factors like tiredness and fatigue. However, the ERP results revealed both reduced early-onset and enhanced late-onset inhibitory activity in the brain (indexed by the Stop N2 and the Stop P3, respectively) as time progressed in the food-reward context relative to the neutral context. These results suggest that despite the absence of a significant time and condition interaction concerning SSRTs, the posttest food-reward block posed a stronger inhibitory challenge. As a consequence, participants displayed an adapted response marked by increased inhibition-related activity in the brain. This adaptive neurophysiological response may reflect the brain’s capacity to dynamically adjust and allocate cognitive resources in response to varying levels of inhibitory demand. Prior studies have provided conflicting results such as increased N2 and P3 amplitudes during food-specific trials (57) and decreased P3 but not N2 in obese participants across all Go/No-go task conditions compared to normal-weight control participants. This suggests that P3 might serve as a more critical biomarker of inhibitory control deficits (58). However, it is important to note that variations in stimuli, paradigms, component timescales, and ERP analyses present challenges in synthesizing results across the existing literature. The diversity in methodologies utilized calls for caution in drawing definitive conclusions from the available evidence. Future research could benefit from standardized protocols and methodologies to address these complexities, enabling more robust comparisons and a deeper understanding of the neurophysiological underpinnings of inhibitory control in various populations.

Despite the valuable insights obtained from this research, several limitations require careful consideration. First of all, it is still controversial whether the DLPFC is a key region of this network [e.g., see Mirabella (59) for a review]. In addition, the right lateralization of the inhibitory network is not a ubiquitous accepted notion (60, 61). Potentially, neither the right nor the left lateralization alone affects inhibitory control (62). Future studies should consider adjusting experimental settings for target regions (63). tDCS involves generating an electric field in the brain tissue, modulating neuronal activity locally and in connected regions, presumably influencing cognitive functions, and ultimately, resulting in behavioral changes. This complex process involves many mediating and confounding variables (64). Additionally, the effectiveness of tDCS depends on numerous stimulation parameters such as duration, intensity, and electrode placement. Although the chosen intervention parameters were based on existing literature and logical reasoning, indeed, alternative stimulation settings might have yielded different outcomes, for example, placing the cathode on extracranial areas rather than the left DLPFC to minimize its impact on the right DLPFC. Our Bayesian analyses indicated that there is support for H_1_ for SSRT. Using a relatively small-sample (*n* = 10) pilot study to identify the effect size of interest can yield extreme results. This can lead to an overestimation of the effect size, which, in turn, can result in an underpowered study. Furthermore, the effect size was calculated for the main effect of tDCS on the primary outcome, FAA, and effects may not translate to the inhibitory indices as captured in the more complex models in the study.

### Conclusions

In our study, we aimed to explore the association between frontal alpha asymmetry and behavioral and brain activity indices of reactive inhibitory control in food-reward contexts. In addition, we assessed the effect of modulation of frontal alpha asymmetry via transcranial direct current stimulation. The results indicated that transcranial direct current stimulation did not yield significant effects on frontal alpha asymmetry. However, we made a notable discovery: There was a dissociation between baseline frontal alpha asymmetry and brain activity indices of inhibitory control. Furthermore, the stop-signal reaction times consistently increased over time, suggesting reduced inhibitory control. Intriguingly, we also observed first a simultaneous decrease and then an increase in inhibitory control-related brain activities, particularly in the food-reward condition. This finding probably indicates a dynamically adaptive response in the presence of food stimuli. Consequently, although the transcranial direct current stimulation yielded negative outcomes, our study reveals that frontal alpha asymmetry still holds the potential to predict neural responses associated with within-task events. Therefore, the study underscores the dynamic relationship between frontal alpha asymmetry and reactive inhibitory processes.

## DATA AVAILABILITY

Data will be made available upon reasonable request.

## SUPPLEMENTAL MATERIALS

10.17605/OSF.IO/R6WNC Supplemental Materials: https://doi.org/10.17605/OSF.IO/R6WNC.

## GRANTS

This work was funded by the Hungarian National Research, Development, and Innovation Office (https://nkfih.gov.hu; grant no. K131635). This research was supported by the ANR Grant awarded within the framework of the Inserm CPJ (ANR-22-CPJ1-0042-01) (to D.N.) and National Brain Research Program by the Hungarian Academy of Sciences (project NAP2022-I-1/2022) (to D.N.).

## DISCLOSURES

No conflicts of interest, financial or otherwise, are declared by the authors.

## AUTHOR CONTRIBUTIONS

A.M.A., R.C., T.N., Z.D., D.N., and H.N.A.L. conceived and designed research; A.M.A. performed experiments; A.M.A. and H. N.A.L. analyzed data; A.M.A., R.C., T.N., Z.D., D.N., and H.N.A.L. interpreted results of experiments; A.M.A. prepared figures; A.M.A. drafted manuscript; R.C., T.N., Z.D., D.N., and H.N.A.L. edited and revised manuscript; A.M.A., R.C., T.N., Z.D., D.N., and H.N.A.L. approved final version of manuscript.
